# Optimizing risk factors to guide COST‐effective use of palivizumab in KOREAN infants

**DOI:** 10.1111/ped.70021

**Published:** 2025-04-11

**Authors:** Ji‐Man Kang, Xavier Carbonell‐Estrany, Bosco Paes, Barry Rodgers‐Gray, John Fullarton, Jean‐Eric Tarride, Hyeon‐Jong Yang, Yun Sil Chang, Ian Keary

**Affiliations:** ^1^ Department of Pediatrics, Severance Children's Hospital Yonsei University College of Medicine Seoul South Korea; ^2^ Institute for Immunology and Immunological Diseases, Yonsei University College of Medicine Seoul South Korea; ^3^ Hospital Clinic Barcelona Spain; ^4^ McMaster University Hamilton Ontario Canada; ^5^ Violicom Medical Limited Aldermaston UK; ^6^ St Joseph's Healthcare Hamilton Ontario Canada; ^7^ SCH Biomedical Informatics Research Unit Soonchunhyang University Seoul Hospital Seoul South Korea; ^8^ Department of Pediatrics, Samsung Medical Center Sungkyunkwan University School of Medicine Seoul South Korea

**Keywords:** cost‐analysis, international risk scoring tool, palivizumab, prophylaxis, RSV

## Abstract

**Background:**

Korean infants born at 32–35 weeks gestational age (wGA) receive palivizumab prophylaxis to prevent respiratory syncytial virus hospitalization (RSVH) if they are born during the RSV season and have a sibling. The aim of this study was to evaluate the impact of using the International Risk Scoring Tool (IRST) to target prophylaxis in Korea.

**Methods:**

The IRST includes 3 risk factors: birth 3 months before to 2 months after the RSV season starts; smokers in the household and/or smoking while pregnant; and, siblings/daycare. First, the accuracy of the Korean guidelines to predict RSVH was compared to that of the IRST using a historic dataset of 13,475 infants born 32–35 wGA. Second, a published cost‐utility model was adapted using Korean‐specific parameters for costs (2022) and resource use to assess the cost‐effectiveness of palivizumab versus no prophylaxis guided either by the Korean guidelines or the IRST.

**Results:**

Using the Korean guidelines identified 26.9% of RSVHs, with an area under the receiver operating characteristic curve of 0.512. The corresponding results for infants assessed at moderate‐ to high‐risk by the IRST were 85.1% and 0.773, respectively. The incremental cost *per* quality‐adjusted life year (QALY) for prophylaxis versus no prophylaxis was ₩29,674,102 (USD22,977) using the Korean guidelines, with a 67.0% probability for cost‐effectiveness against a willingness‐to‐pay threshold of ₩41,655,203 (USD32,255). For the IRST, it was ₩26,265,142 (USD20,338)/QALY and 70.8% probability.

**Conclusions:**

Adoption of the IRST in Korea would provide greater protection of the most vulnerable infants born 32–35 wGA against RSVH whilst improving cost‐effectiveness.

## INTRODUCTION

Respiratory syncytial virus (RSV) is a major childhood pathogen causing almost 1.7 million acute respiratory infections (ARIs) and over 400,000 hospitalizations (RSVH) in children younger than 5 years old in high‐income countries every year.^1^ In Korea, RSV is recognized as the most common cause of community‐acquired pneumonia requiring hospitalization in children <2 years.^2^, ^3^ Studies have reported that RSV accounts for up to 30% of all ARIs and 53% of all viral ARIs in Korean children <5 years.^4^ Korea has a temperate climate and the RSV season typically spans autumn through early spring (October to March), placing increased pressure on pediatric services during these months.

Palivizumab, a humanized immunoglobulin G‐1 monoclonal antibody that binds to the F‐protein of RSV, has proven effective in preventing severe RSV infection in high‐risk infants, including those with bronchopulmonary dysplasia (BPD) or congenital heart disease (CHD) and those born prematurely at ≤35 week's gestational age (wGA).^5^, ^6^ Palivizumab was first marketed in Korea in 2005 and remains the only approved RSV‐targeting preventive measure available in the country. The current guidelines from the Korean National Health Insurance Service, enacted in 2016, recommend palivizumab for children <2 years old with BPD and haemodynamically significant CHD and infants ≤6 months of age at the onset of the RSV season if born at <32 wGA. For infants born at 32–35 wGA, the guidelines recommend the use of palivizumab only if the infant is born during the RSV season and has one or more siblings or older step‐siblings.

Proximity of birth to the RSV season and the presence of siblings are both well‐established risk factors associated with an increased risk of RSVH in 32–35 wGA infants.^7^, ^8^, ^9^, ^10^, ^11^, ^12^ Importantly, however, it was reported in a pooled analysis of seven studies encompassing data from 14,504 infants born 32–35 wGA that 42.3% of RSVHs occurred in those born outside of the RSV season.^13^ Moreover, with growing uncertainty around the seasonal circulation of RSV post‐COVID‐19, including reports of increased off‐season RSV activity and delayed onset of the RSV season,^14^ limiting prophylaxis solely to 32–35 wGA infants born in season may no longer be the most effective strategy. Similarly, when considering siblings, declining birth rates in Korea and increasing numbers of single‐child families raise the possibility of diminishing importance of this sole risk factor to guide prophylaxis in 32–35 wGA infants.^15^ Consideration of a wider range of risk factors might enable more effective targeting of prophylaxis in these moderate‐to‐late preterm infants.

**FIGURE 1:**
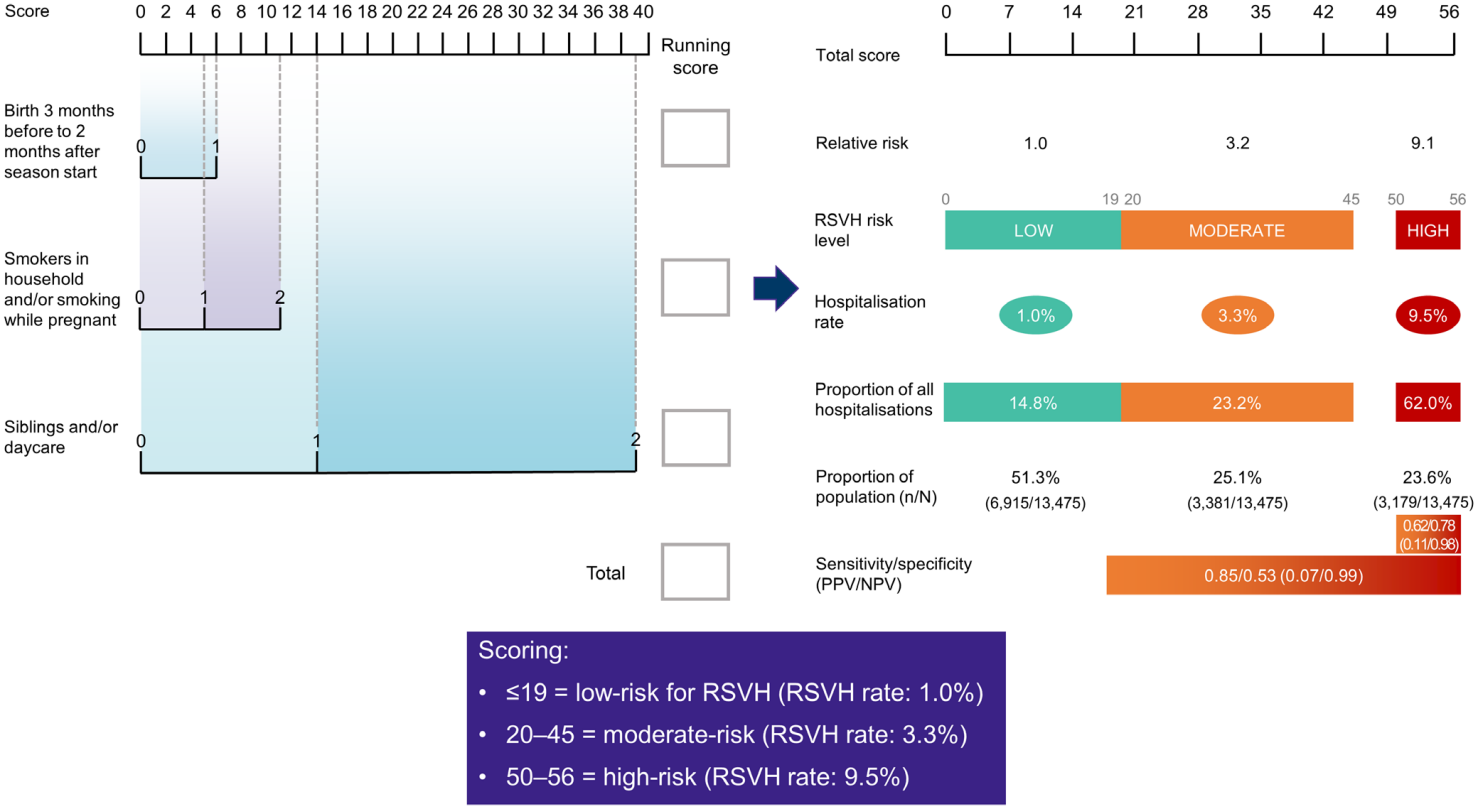
Interpretation of the IRST risk score and risk group characteristics (adapted from^16^). IRST, International Risk Scoring Tool, NPV, Negative predictive value; PPV, Positive predictive value; RSV, Respiratory syncytial virus; RSVH, RSV hospitalization.

The International Risk Scoring Tool (IRST) was developed using data on 13,475 infants (484 [3.6%] with RSVH) born 32–35 wGA from six observational studies across the northern hemisphere and comprises three composite risk factors: (i) birth between 3 months before and 2 months after RSV season start date; (ii) smokers in the household and/or maternal smoking whilst pregnant; and, (iii) siblings (excluding multiple births) and/or daycare attendance (Figure 1).^16^ The IRST assigns infants at either low‐(score ≤ 19; 1.0% risk of RSVH), moderate‐(20–45; 3.3%), or high‐risk (50–56; 9.5%) risk of RSVH and was externally validated against the Irish PREMI (RSV Preterm Risk Estimation Measure for RSVH in Ireland) study^12^, ^16^ and using data from Colombia^17^ and Brazil.^18^ Importantly, the IRST has been demonstrated to guide palivizumab prophylaxis cost‐effectively within the Canadian,^19^ Italian,^20^ and Colombian healthcare systems.^21^

The aim of this study was to assess the potential impact of utilizing the IRST rather than the current Korean guidelines to target prophylaxis against RSVH in infants born 32–35 wGA.

## METHODS

### Prediction of RSVH and implications for prophylaxis

Several analyses were undertaken to explore how the Korean guidelines and the IRST compare in identifying infants born at 32–35 wGA who are at the greatest risk of RSVH. Korean birth statistics^15^ for the 10‐year period 2013–2022 were analyzed to confirm and quantify the trend of a declining number of infants having siblings. An analysis was then undertaken using the pooled dataset that was employed to develop the IRST^16^ (Table S1) to quantify the relationship between the number of siblings and RSVH rate. The predictive potential of being born in season to a family with sibling(s) (Korean guidelines) was calculated in the pooled dataset using logistic regression, where RSVH was the dependent variable and the risk factors were the covariates, and the results compared to that of the original IRST. Predictive accuracy was assessed by calculation of the area under the receiver operating characteristic curve (AUROC), with a result of ≥0.75 considered “good.”^22^ How predictive accuracy relates to RSVH risk and the proportion of the population to be prophylaxed was also calculated. Finally, an assessment was made to characterize the infants who would be identified at moderate or high risk of RSVH according to the IRST (and thereby eligible for prophylaxis) but would be missed if only born in season and sibling(s) were considered.

### Cost‐effectiveness of palivizumab versus no prophylaxis

**FIGURE 2:**
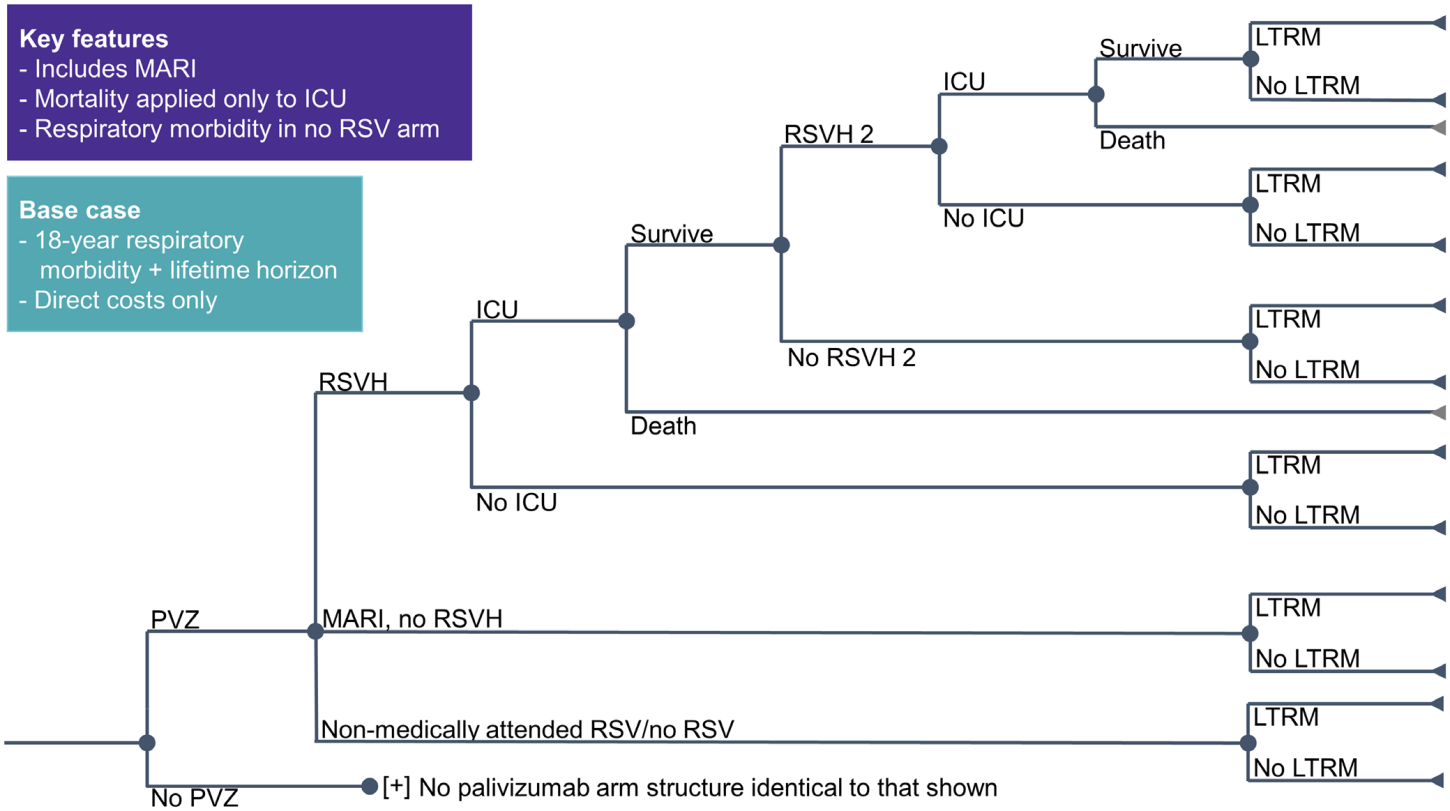
Decision tree for cost‐utility model (adapted from^19^). ICU, Intensive care unit; LTRM, Long‐term respiratory morbidity; MARI, Medically attended RSV infection; PVZ, Palivizumab; RSVH, Respiratory syncytial virus hospitalization.

A previously published cost‐utility model,^19^ incorporating a decision tree (Figure 2), was adapted for the Korean setting using country‐specific parameters for resource use (Table S2) and costs (Table S3), where available. Two versions of the model were developed: (1) Where infants received palivizumab prophylaxis if they were born in the RSV season and had sibling(s), as *per* the Korean guidelines; and, (2) Where infants received palivizumab prophylaxis if scored at moderate or high risk of RSVH by the IRST. In both versions, infants experienced either RSVH, emergency department (ED)/outpatient medically attended RSV infection (MARI), or remained uninfected/non‐attended.

RSVH rates were predicted using the pooled database (*N* = 13,475) that generated the IRST.^16^ Palivizumab efficacy at reducing RSVH in moderate‐to‐late preterm infants was 82.2%, taken from the IMpact‐RSV trial.^23^ Mortality (0.43%)^24^, ^25^ was applied only to the 4.15% (Yang H‐J, 2023, unpublished data) of infants admitted to the intensive care unit (ICU) following RSVH. A MARI rate of 2.95%, among infants >32–35wGA in the palivizumab arm of the motavizumab trial,^26^ was assumed for infants receiving prophylaxis and adjusted using data from the US REPORT (Respiratory Events Among Preterm Infants Outcomes and Risk Tracking) study^11^ to calculate the proportion presenting to outpatients only (2.48%), ED only (0.05%), or both (0.42%). For infants not receiving prophylaxis, MARI rates were calculated by uplifting the palivizumab rates by removing the efficacy observed in the IMpact‐RSV trial (outpatients only: 13.92%; ED only: 0.29%; both: 2.36%;).^11^, ^23^, ^26^

Respiratory morbidity was modeled up to 18 years of age in infants following RSVH or MARI and up to 6 years of age in uninfected/non‐medically attended infants (Table S4).^27^, ^28^, ^29^, ^30^ Disutility for RSVH and respiratory morbidity were included in the model (Table S2).^19^ Prophylaxis costs were calculated using Korean prices, published birthweights,^31^ and a growth algorithm.^19^, ^32^ The average number of palivizumab doses *per* infant was calculated by assuming an even spread of births across the year, an RSV season running from October to March, and 100% compliance to a maximum of five doses in line with the palivizumab label. A 4.5% discount was applied as *per* Korean guidelines for pharmacoeconomic evaluations.^33^

The model adopted the healthcare provider perspective over a lifetime horizon with outcomes expressed as the cost *per* quality‐adjusted life year (QALY) for palivizumab versus no prophylaxis. Probabilistic (PSA; 10,000 iterations) and deterministic (DSA) sensitivity analyses, employing limits of plus or minus 10% (if no measures of dispersion available) and 20% on the values of the tested variables, respectively, were conducted to assess uncertainty. The PSA was assessed against a willingness‐to‐pay (WTP) threshold of ₩41,655,203 (USD32,254.60); the gross domestic product per capita of Korea. An alternative scenario from a societal perspective (indirect costs included) was explored (Table S3).

All analyses were performed using SPSS for Windows version 15.0 (IBM Corporation), Microsoft Access SQL 365, and Microsoft Access/Excel VBScript 365 (Microsoft Corporation).

### Ethical approval

This study analyzed data from previously published studies and, therefore, did not require ethical approval or informed consent.

## RESULTS

### Prediction of RSVH and implications for prophylaxis

#### Korean birth statistics

**FIGURE 3:**
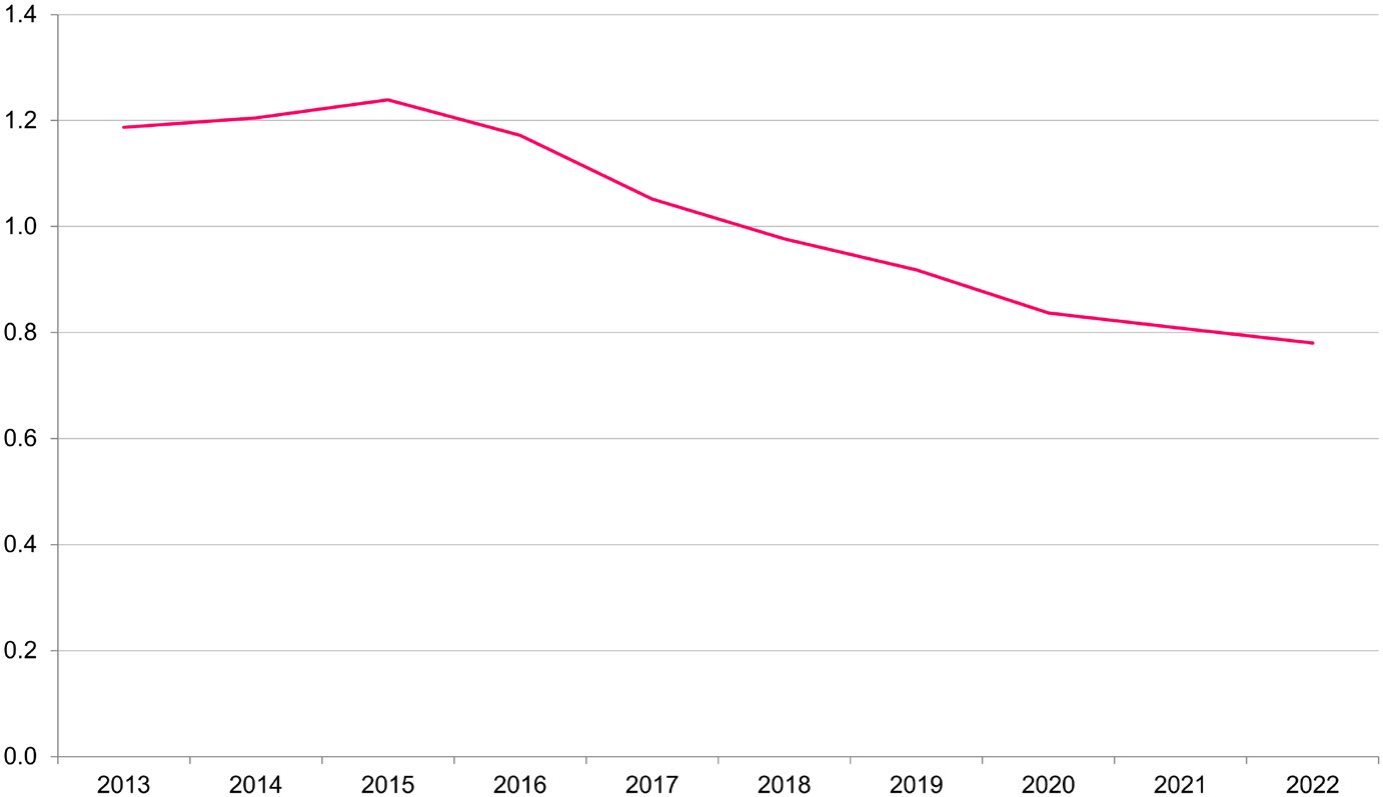
Fertility rate in Korea over 10 years between 2013 and 2022.

The Korean fertility rate has fallen gradually over the last 10 years from 1.2 in 2013 to 0.78 in 2022 (Figure 3). Using data from 2020, it is evident that the number of single‐child households has increased over time, with 9.5% of mothers ≥60 years having a single child, rising to 24.9% in mothers aged 40–49 years. The average age of primigravidae in 2022 was 33.5 years.

#### Importance of siblings as a risk factor

**FIGURE 4:**
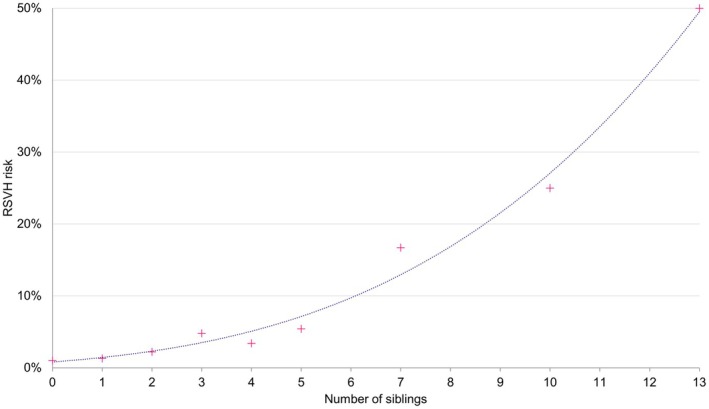
The relationship between number of siblings and RSVH risk using the IRST pooled dataset (*N* = 13,475). IRST, International Risk Scoring Tool; RSV, Respiratory syncytial virus; RSVH, RSV hospitalization.

Analysis of the pooled dataset underlying the IRST^16^ revealed an apparent strong positive correlation between the number of siblings and RSVH risk (Figure 4). For infants without a sibling, the RSVH rate was 1%, which rose to 1.5% with the presence of 1–2 siblings, and 5.1% for ≥3 siblings. In the IRST, the presence of siblings was the most powerful risk factor alongside daycare attendance, with a score of 14 (low risk) when one of these variables was present, and 39 (moderate risk) when both variables were combined (Figure 1).^16^ The adjusted risk of RSVH associated with the presence of sibling(s) within the IRST produced an odds ratio of 1.6 (95% confidence interval [CI] 1.4–2.0; *p* < 0.001).^16^

#### Predictive ability

**FIGURE 5:**
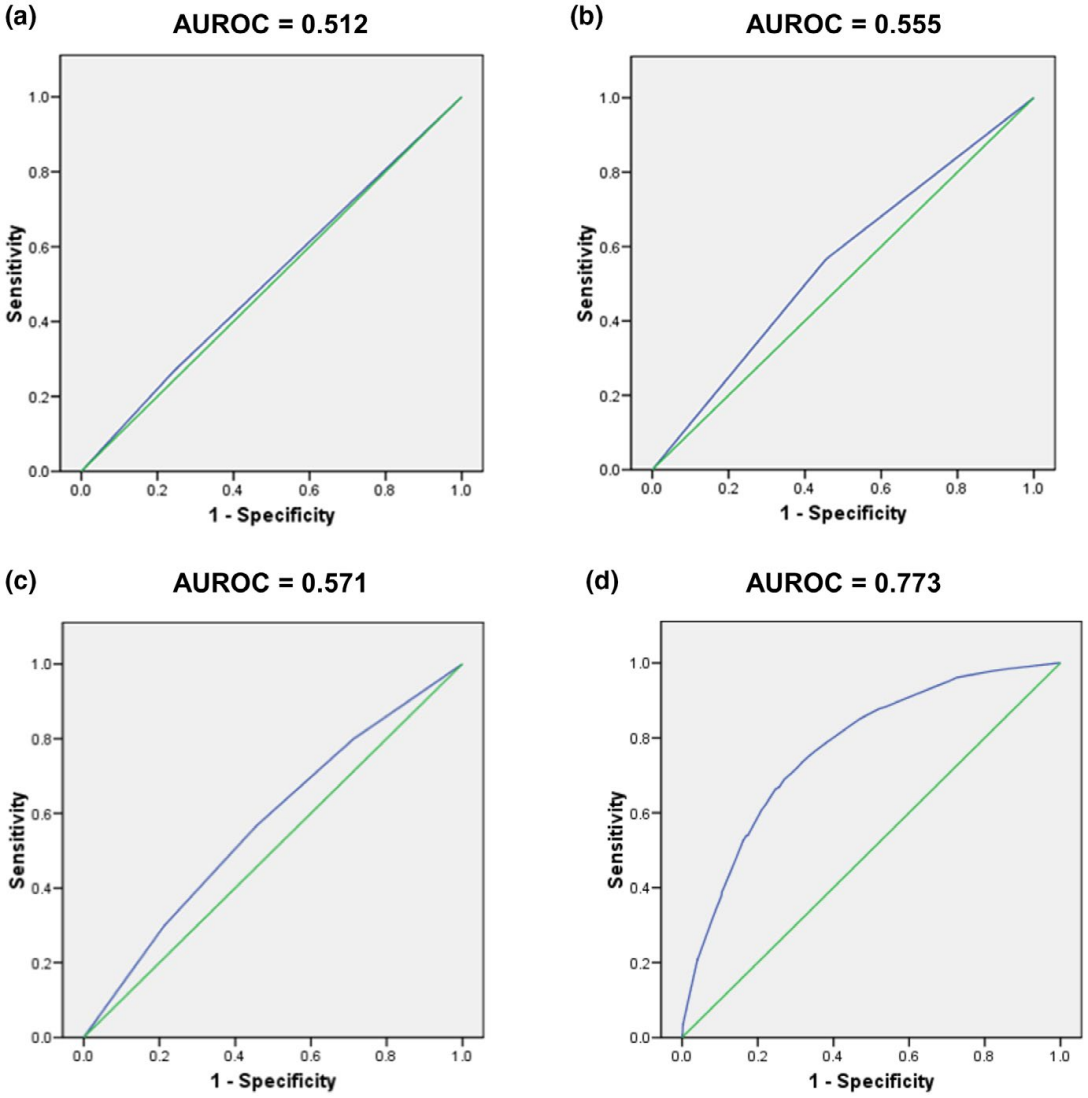
The predictive accuracy of birth in the RSV season with siblings (a), siblings alone (b), birth in the RSV season and/or siblings (c) compared to the IRST (d). AUROC, Area under the receiver operating characteristic curve, where 1 represents perfect predictive accuracy and 0.5 no better than chance; IRST, International Risk Scoring Tool.

Using birth during the RSV season plus the presence of sibling(s) as a combined variable (commensurate with the Korean guidelines) identified 26.9% (130/484) of RSVHs in the pooled dataset with an AUROC of 0.512 (Figure 5a). If sibling(s) was used as a standalone risk factor, 56.6% (274/484) of RSVHs were correctly identified with an improved AUROC of 0.555 (Figure 5b). Similarly, if birth in the RSV season was used on its own, 46.7% (226/484) RSVHs were correctly identified with an AUROC of 0.580. When the risk factors for birth in the RSV season and the presence of sibling(s) were applied separately, an AUROC of 0.571 was generated (Figure 5c; of note an agreed cut‐off would need to be ascertained to determine risk and, thereby, number/percentage of RSVHs covered). By comparison, the IRST correctly identified 85.1% (412/484) of RSVHs and had an AUROC of 0.773 (Figure 5d).^16^ For the age variable in the IRST, birth 3 months before to 2 months after the RSV season start date, this risk factor alone correctly identified 57.9% (280/474) of RSVHs.

#### Proportion of population to receive prophylaxis and RSVH risk

Under the Korean guidelines, 24.5% (3296/13,475) of infants would receive prophylaxis to prevent a potential 26.9% (130/484) of RSVHs. The corresponding results for the IRST were 48.7% (6560/13,475) to prevent 85.1% (412/484) of RSVHs. Infants meeting the Korean guidelines criteria had an RSVH risk of 3.9%, whilst those deemed at moderate‐ and high‐risk by the IRST had a 6.3% risk (these rates were used in the cost‐utility analysis).

#### Characterization of infants at moderate or high risk of RSVH using the IRST that would be missed using birth in the RSV season plus sibling(s)

Excluding the criteria of being born during the RSV season and having siblings, a total of 12 combinations of the risk factors within the IRST scored infants at a moderate or high risk of RSVH and, thereby, eligible for prophylaxis (Table 1). Seven of these 12 combinations include infants born ≤3 months before the start of the RSV season, nine included smoking (maternal and/or household), and all included siblings and/or daycare.

**TABLE 1:** Infants scored at moderate or high risk of RSVH by the IRST without being born in the RSV season and having a sibling(s).

Birth 3 months before to 2 months after RSV season start^a^ (6)	Smokers in the household/or smoking while pregnant (5)	Smokers in the household and smoking while pregnant (11)	Daycare (14)	Siblings (14)	Daycare and siblings (39)	Risk score and category
+	−	−	+	−	−	20–Moderate
+	−	−	−	+	−	20–Moderate
−	N/A	+	+	−	−	25–Moderate
+	+	−	+	−	−	25–Moderate
−	N/A	+	−	+	−	25–Moderate
+	+	−	−	+	−	25–Moderate
+	N/A	+	+	−	−	31–Moderate
+	N/A	+	−	+	−	31–Moderate
−	−	−	N/A	N/A	+	39–Moderate
−	+	−	N/A	N/A	+	44–Moderate
−	N/A	+	N/A	N/A	+	50–High
+	N/A	+	N/A	N/A	+	56–High

Abbreviations: IRST, International Risk Scoring Tool; N/A, Not applicable; RSV, respiratory syncytial virus; RSVH, RSV hospitalization.

^a^ When used in combination with siblings, this assumes that the infant was born ≤3 months before the start of the RSV season.

### Cost‐utility model

The average cost of palivizumab in the base case was estimated to be ₩3,855,828 (USD2,986; including nurse administration of the monoclonal antibody and an average number of injections of 4.09; see footnote to Table S3 for more details of calculation). The incremental cost *per* QALY (ICUR) for palivizumab (versus no prophylaxis) was ₩29,674,102 (USD22,977) when using birth within the RSV season plus siblings and ₩26,265,142 (USD20,338) for infants scored at moderate‐ and high‐risk for RSVH by the IRST (Table 2).

**TABLE 2:** Cost‐effectiveness of palivizumab prophylaxis directed by the Korean guidelines (born in the RSV season plus sibling(s)) or the IRST (versus no prophylaxis).

	Infants receiving prophylaxis	
	Korean guidelines (born in RSV season plus siblings)	Moderate and high risk according to IRST
Difference in costs	₩3,612,183	₩3,573,521
Difference in QALYs	0.122	0.136
Cost per QALY	₩29,674,102	₩26,265,142

Abbreviations: IRST, International Risk Scoring Tool; QALY, quality‐adjusted life year; RSV, respiratory syncytial virus; ₩, Korean won.

**FIGURE 6:**
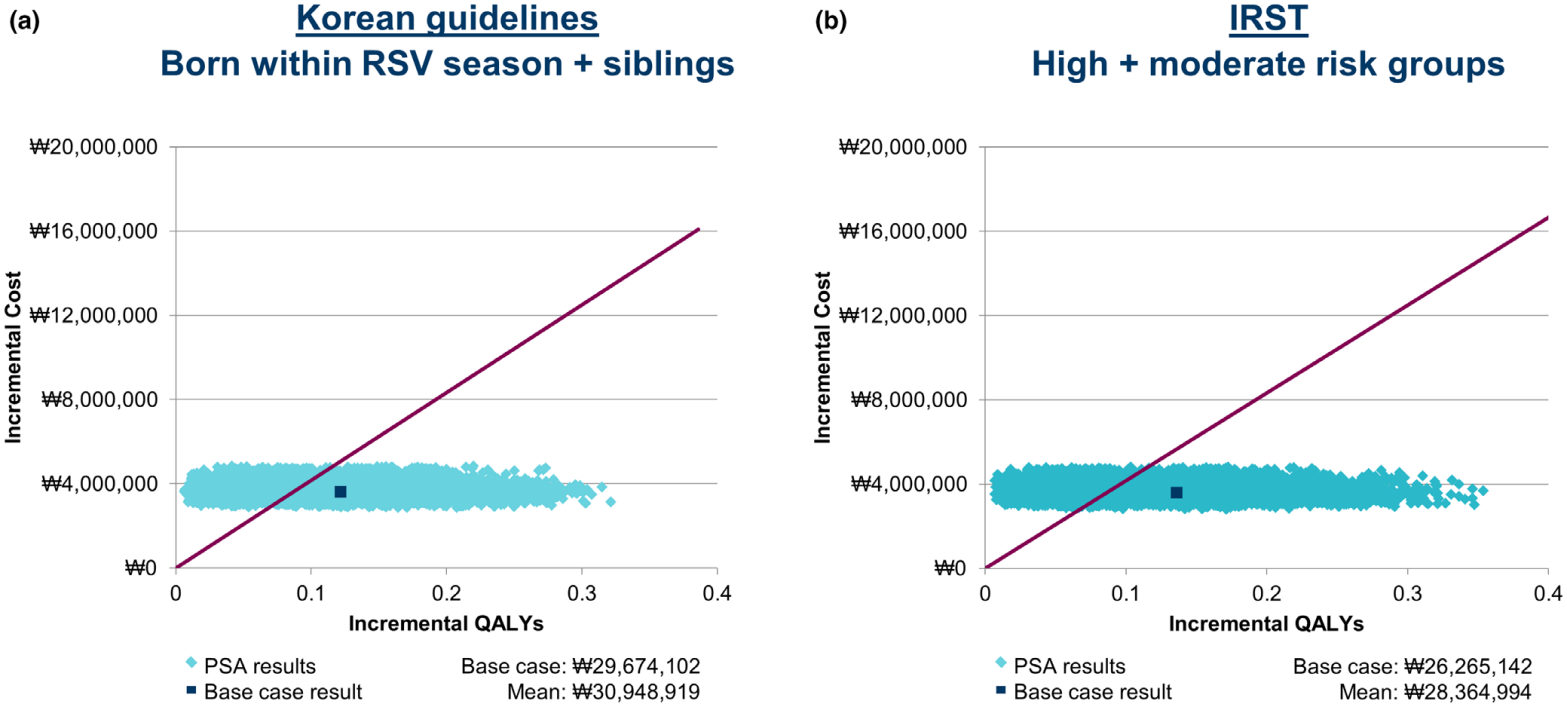
Incremental cost‐effectiveness plane for palivizumab versus no prophylaxis (₩41,655,203 WTP threshold) for Korean guidelines‐(birth in the RSV season plus siblings) guided prophylaxis (a) and for IRST‐guided prophylaxis (b). 18 years morbidity with lifetime horizon; 10,000 iterations; assumed 10% variance if no measure of dispersion; PSA, Probabilistic sensitivity analysis; QALY, Quality‐adjusted life year; RSV, Respiratory syncytial virus; WTP, Willingness‐to‐pay.

The PSA resulted in a 67.0% probability of prophylaxis being within the WTP cost‐effectiveness threshold (₩41,655,203 [USD32,255]) when directed by the Korean guidelines (Figure 6a) versus a 70.8% probability for IRST‐guided prophylaxis (Figure 6b). The DSA found both versions of the model (Korean guidelines and IRST) to be most sensitive to utility scores, rate of long‐term respiratory morbidity, palivizumab efficacy, and palivizumab cost (Figure S1a,b).

The inclusion of indirect costs resulted in a cost/QALY for palivizumab (versus no prophylaxis) of ₩30,638,265 (USD23,724) when using birth within the RSV season plus siblings and ₩26,935,744 (USD20,857) for infants scored moderate‐ and high‐risk for RSVH by the IRST. Both results were marginally higher than the base case.

## DISCUSSION

Moderate‐to‐late preterm infants (born 32–35 wGA) are well‐recognized to be at increased risk of RSVH.^7^, ^8^, ^9^, ^10^, ^11^, ^12^, ^13^ Identification of those 32–35 wGA infants with additional risk factors that increase the likelihood of RSVH can help guide the clinical‐ and cost‐effective use of palivizumab. Since 2016, Korean guidelines have recommended the use of palivizumab in 32–35 wGA infants if they are born during the RSV season and have one or more siblings. Whilst both are established risk factors,^7^, ^8^, ^9^, ^10^, ^11^, ^12^, ^13^ analysis of a large international dataset indicates that these criteria identify only 26.9% of potential RSVHs in these infants due to a lack of discriminatory power (AUROC 0.512). Utilization of these two risk factors as separate variables (as an “and/or” construct) improved prediction; however, the AUROC (0.571) still fell below the threshold of ≥0.75 generally considered acceptable for a risk scoring tool.^22^ By contrast, the three variable (five risk factor) IRST identified 3‐fold more potential RSVHs (85.2%) with a high degree of predictive accuracy (AUROC 0.773).^16^

Both the Korean guidelines and IRST include siblings as a variable for the prediction of RSVH. However, the falling birth rate in Korea and increasing number of single‐child families appear to diminish the influence and value of this risk factor as a predictor for RSVH. Analysis of Korean population statistics from 2020 found that at least 25% of children will likely have no siblings and, thereby, be excluded from receiving prophylaxis under the current guidelines. With fertility decreasing annually over the last decade in Korea (from 1.2 in 2013 to 0.78 in 2022), this proportion is likely to increase. The analysis also revealed that, as to be expected when considering viral transmission, the risk of RSVH is strongly correlated with the number of siblings, rising from 1% for 32–35 wGA infants without a sibling, to 5.1% for those with ≥3 siblings. Hence, with children having fewer siblings, the importance or weighting of this risk factor to identify infants at increased risk of RSVH will decrease commensurately. Importantly, the wider number of risk factors comprising the IRST allows identification of infants eligible for prophylaxis even when excluding siblings and birth within the RSV season, with 12 such combinations scoring infants at moderate or high risk of RSVH. This included utilization of smokers in the household and/or smoking while pregnant, which were present in nine of the 12 combinations. Both smoking variables are well‐established as significant risk factors for childhood RSVH^34^ and, importantly, are modifiable. Encouragingly, smoking rates appear to be declining in Korea, albeit they remain higher in males than females (30% vs 5%, respectively, in 2022).^35^ For those that do smoke while pregnant and/or in the household, then, based on global data,^16^, ^34^ it is reasonable to assume an increased risk of RSVH for their infant and that smoking should be considered in any RSV risk assessment tool or prophylaxis guidelines for 32–35 wGA infants in Korea.

Within the IRST, siblings are paired with daycare attendance (this being the most powerful combination of risk factors within the tool) and it is interesting to note that the enrolment rate of Korean children in preschools has steadily increased over the past 10 years, from 47.9% in 2013 to 53.4% in 2022.^36^ Thus, it might be postulated that daycare attendance is becoming an increasingly important risk factor for RSV transmission and hospitalization in Korea. This perhaps also supports the logic of including both daycare attendance and siblings as part of a rigorously selected risk factor model predicting RSVH in moderate‐to‐late preterm infants.^37^

When considering the age variable in the Korean guidelines (birth in season) and the IRST (birth 3 months before to 2 months after the RSV season start date) as individual risk factors, the latter correctly identified an additional 11.2% of RSVHs (46.7% vs 57.9%, respectively). This is perhaps unsurprising since infants <6 months old before the RSV season are a well‐recognized high‐risk group^7^, ^8^, ^9^, ^10^, ^11^, ^12^ and the chronological age range for the IRST variable was specifically refined to optimize the identification of RSVHs by maximizing predictive power.^16^ This is illustrated in the analysis excluding the combination of birth in season and siblings, wherein seven of the 12 combinations of IRST risk factors scoring infants at moderate or high risk of RSVH included those born ≤3 months before the start of the RSV season (Table 1).

Although the IRST demonstrates several salient advantages over the current Korean guidelines in terms of predictive accuracy, the identification of more infants eligible for prophylaxis with the former than the latter (48.7% vs 24.5% of the population, respectively) has implications for the cost‐effectiveness of palivizumab. In this first analysis of the cost‐effectiveness of palivizumab in Korean moderate‐to‐preterm infants, prophylaxis was found to be highly cost‐effective (against a WTP threshold of ₩41,655,203 [USD32,255]) when directed by criteria either in the current Korean guidelines or the IRST. However, IRST‐guided prophylaxis was found to be the most cost‐effective approach (₩26,265,142 [USD20,338] vs ₩29,674,102 [USD22,977] with the Korean guidelines). Hence, despite an increased number of infants being eligible for prophylaxis under the IRST than the Korean guidelines, this is more than offset by the differential in predictive accuracy.

The main limitation of this study was the availability of Korean‐specific data on RSVH in infants born 32–35 wGA, as the pooled dataset (*N* = 13,475) underpinning the IRST that was used in the present analyses included data from only 131 Korean infants (as part of the PONI study).^9^, ^10^ Whilst the pooled dataset included data from 28 culturally and regionally diverse countries, all, including Korea, are part of the northern temperate zone and have similar RSV seasonality. Furthermore, analysis has found that the risk factors identified in the individual studies and the pooled dataset appear applicable and universal across all of the included countries.^9^, ^10^, ^37^ Hence, it can be surmised that the data used in this analysis are representative of Korea, in the absence of any local, country‐specific data. It could also be argued that the pooled dataset represents the best case for assessing the Korean guidelines since with the falling birth rate, fewer infants would have siblings and, therefore, be identified as being at increased risk of RSVH. A further limitation of the study was the absence of Korean data sources describing the short‐ and long‐term quality of life outcomes for infants born at 32–35 wGA. However, this is a generally recognized data gap for cost‐utility models of preventive strategies for RSV in children.^19^, ^20^, ^21^, ^38^

## CONCLUSION

Consideration should be given to the application of the IRST in routine clinical practice in Korea as it has the potential to provide more accurate and cost‐effective targeting of palivizumab prophylaxis to the most vulnerable moderate‐to‐late preterm (32–35 wGA) infants. Implementation of this strategy will likely reduce the overall burden of severe RSV infection in this population.

## AUTHOR CONTRIBUTIONS

JMK, IK, BP, BRG, JF, and XCE designed the study. JMK, HJY, and YSC provided and verified Korean data for inclusion in the cost‐utility analysis. IK and JF undertook the analyses. JET provided technical expertise on the cost‐utility analysis. BRG drafted the manuscript and all authors provided feedback/edits and approved the final version.

## CONFLICT OF INTEREST STATEMENT

BP and XCE have received research funding and/or compensation as advisor/lecturer from AstraZeneca and Sanofi. BRG, IK, and JF employers have received payment from AstraZeneca for work on various projects. JMK, JET, HJY, and YSC have nothing to declare.

## Supporting information


Data S1.
