# Supplementary material for: Optimizing risk factors to guide COST‐effective use of palivizumab in KOREAN infants
Source: Pediatr Int. 2025 Apr 11;67(1):e70021. doi: 10.1111/ped.70021 (PMC11987582; doi:10.1111/ped.70021)
Supplement: Supplementary file 1 — Data S1. [file PED-67-e70021-s001.docx]

**Supporting Information**

**Table S1: Risk factors included in pooled dataset** **(adapted from^[[1]](#endnote-2)^)**

| **Risk factor** | **RSVH**  **(n=484)** | **Non-hospitalised**  **(n=12,991)** | **Odds Ratio (95% CI)** | **p-value** |
| --- | --- | --- | --- | --- |
| Birth 3 months before to 2 months after  RSV season start | 293 (60.5%) | 5,552 (42.7%) | 2.0 (1.7-2.5) | <0.001 |
| Smoking whilst pregnant | 78 (20.7%)^a^ | 1,316 (13.4%)^b^ | 1.7 (1.3-2.2) | <0.001 |
| Siblings at home (excluding twin) | 286 (59.1%) | 6,093 (47.0%)^c^ | 1.6 (1.3-1.9) | <0.001 |
| Child in day care | 107 (22.2%)^d^ | 1,998 (15.5%)^e^ | 1.6 (1.3-1.9) | <0.001 |
| Smokers in household | 183 (37.8%) | 3,941 (30.4%)^f^ | 1.4 (1.2-1.7) | 0.001 |

CI: confidence interval; RSVH: respiratory syncytial virus-related hospitalisation. ^a^n=377; ^b^n=9,846; ^c^n=12,960; ^d^n=482; ^e^n=12,921; ^f^n=12,946.

**Table S2: Input parameters associated with RSVH and MARI**

| **Parameter** | **Point estimates** | | **Source(s)** |
| --- | --- | --- | --- |
|  | **PVZ** | **No PVZ** |  |
| **PVZ efficacy (RRR)** | 82.2% | - | Notario *et al*. 2014^[[2]](#endnote-3)^ |
| **RSVH***  - Overall rate  - IRST moderate + high risk  - Born in RSV season + sibling(s)  - ICU rate  - Ward LOS, mean days  - Utility in hospital  - Utility post discharge  - No sequelae  - Long-term sequelae  - Mortality^✝^ | 1.12%  0.71%  4.15%  5.75  0.60  0.88  0.79  0.43 | 6.3%  3.9%  4.15%  5.75  0.60  0.88  0.79  0.43 | Notario *et al*. 20142 &Blanken *et al*. 2018^1^  Notario *et al*. 20142 & Blanken *et al*. 20181  Personal communication (Prof Yang)  Personal communication (Prof Yang)  Weiner *et al*. 2012^[[3]](#endnote-4)^, Leidy *et al*. 2005^[[4]](#endnote-5)^  Greenough *et al*. 2004^[[5]](#endnote-6)^  Chiou *et al*. 2005^[[6]](#endnote-7)^  Wang *et al*. 2008^[[7]](#endnote-8)^ & 2011^[[8]](#endnote-9)^ |
| **MARI**  - Rate outpatients only  - Rate outpatients & ED  - Rate ED only  - Utility no sequelae  - Utility long-term sequelae | 2.48%  0.42%  0.05%  0.95  0.79 | 13.92%  2.36%  0.29%  0.95  0.79 | Notario *et al*. 20142, Ambrose *et al* 2014^[[9]](#endnote-10)^, Carbonell *et al*. 2010^[[10]](#endnote-11)^  Greenough *et al*. 2004^5^  Chiou *et al*. 2005^6^ |
| **MARI/No RSV^‡^**  - Utility  - No sequelae or respiratory symptoms  - Long-term sequelae or respiratory symptoms | 0.95  0.79 | 0.95  0.79 | Greenough *et al*. 2004^5^  Chiou *et al*. 20056 |
| **Birth weight (g)** | 2536.00 | 2536.00 | Lim *et al* 2014^[[11]](#endnote-12)^ |

*First and subsequent RSVHs; ^✝^Applied only to patients in ICU; ^‡^Infants without an RSV infection or one not requiring medical management. ED: emergency department; ICU: intensive care unit; LOS: length of stay; MARI: medically-attended RSV infection not requiring hospitalisation; PVZ: palivizumab; RRR: relative risk reduction; RSV: respiratory syncytial virus; RSVH: RSV-related hospitalisation.

**Table S3: Direct and indirect costs**

| Parameter | Cost KRW (₩) [USD] | Units | Source(s) |
| --- | --- | --- | --- |
| **Palivizumab^**  - 50 mg vial  - 100 mg vial  - Nurse administration | 535,851.00 [414.92]  922,644.00 [714.42]  4,000.00 [0.32] | Lowest combination of vials *per* infant weight  1 *per* injection | Korean list price  Korean list price  Personal communication (Prof Kang) |
| **Pre-admission healthcare contact** | 214,570.48 [166.15] | 1 *per* RSVH* | Kim *et al*. 2018^[[12]](#endnote-13)^ |
| **RSVH total stay (excluding ICU)** | 662,835.67 [513.25] | 1 *per* RSVH* | Kim *et al*. 201812 & Personal communication (Prof Yang) |
| **ICU** | 15,874,170.69 [12,291.74] | 1 *per* ICU admission | Personal communication (Prof Yang) |
| **MARI**  - Outpatient visit  - Outpatient plus ED  - ED visit  - Follow-up appointment | 71,523.49 [55.38]  286,093.97  [221.53]  214,570.48 [166.15]  71,523.49 [55.38] | 1 *per* affected infant* | Kim et al. 201812 |
| **Respiratory morbidity (*per* annum)** ^✝^ | 181,566.60 [140.59] | 1 *per* affected infant^‡^ | Park *et al*. 2006^[[13]](#endnote-14)^ |
| **Societal (indirect) costs** | | | |
| **Palivizumab administration**  - Transport  - Missed work | 63,804.00 [49.40]  105,120.87  [81.40] | 1 *per* infant receiving palivizumab | Based on cost of taxi fare within Seoul assuming a 20km round trip *per* injection^[[14]](#endnote-15)^  Statistics Korea (salary),^[[15]](#endnote-16)^ assumes 3 hour missed *per* injection for 50% of injections |
| **RSVH**  - Missed work  - Childcare**  - Transport**  - Other out of pockets** | 73,3361.54 [567.86]  116,509.32 [90.22]  125,364.03 [97.07]  343,189.85  [265.74] | 1 *per* infant with RSVH | Mitchell *et al*. 2017^[[16]](#endnote-17)^ & Statistics Korea (salary)15  Mitchell *et al*. 201716  Mitchell *et al*. 201716  Mitchell *et al*. 201716 |
| **MARI attendance**  - Transport  - Missed work | 15,600.00 [12.08]  25,701.92 [19.90] | 1 *per* infant with MARI | Based on cost of taxi fare within Seoul assuming a 20km round trip *per* injection14  Statistics Korea (salary),15  assumes 3 hours *per* 50% of MARI attendance |
| **Loss of earnings following death** | 1,198,002,960.00  [927,641.77] | 1 *per* infant suffering mortality | Statistics Korea (salary)15 & OECD (employment & years lost productivity)^[[17]](#endnote-18)^ |

*See Supporting Table 1 for rates; ^✝^Costs adjusted to 2023 Korean won levels using World Bank data, with USD1=KRW1,291.45^[[18]](#endnote-19)^; ^‡^see Supporting Table 3 for rates; **: Costs converted from Mitchell et al using Statistics Canada CPI data^[[19]](#endnote-20)^ and Purchasing Price Parities;^[[20]](#endnote-21)^ ^The cost of palivizumab was calculated using the Korean price (50 mg: ₩535,851 [USD414.92]; 100 mg: ₩922,644 [USD714.42]) and the lowest combination of vials per infant weight, assuming no vial sharing, and 100% compliance. Using the average birthweight of a Korean 32–35 wGA infant (2,536 g)^11^ and assuming an even spread of births across the year, infant weight at palivizumab administration was predicted using the growth algorithm described by Narayan et al. 2020,^[[21]](#endnote-22)^ assuming an RSV season from October to March. The average number of palivizumab doses was calculated similarly by assuming an even spread of births across the year, an RSV season from October to March and 100% compliance to a maximum of five doses in line with the palivizumab label. Predicated on this approach, the average cost of palivizumab was estimated to be ₩3,855,828 (USD2,986) *per* infant, when nurse administration was included, with an average number of injections required of 4.09. ED: emergency department; ICU: intensive care unit; KRW: Korean won; MARI: medically-attended RSV infection; OECD: Organisation for Economic Cooperation and Development; RSV: respiratory syncytial virus; RSVH: RSV-related hospitalisation

**Table S4: Rates of long-term respiratory morbidity**

| **Year** | **Palivizumab** | | **No Palivizumab** | | **Source(s)** |
| --- | --- | --- | --- | --- | --- |
|  | **RSVH** | **No RSVH** | **RSVH** | **No RSVH** |  |
| **0–1** | 18.4% | 5.4% | 41.4% | 12.1% | Respiratory morbidity from SPRING study (Carbonell-Estrany *et al*. 2015^[[22]](#endnote-23)^), as modified by Sanchez Luna *et al*. 2017^[[23]](#endnote-24)^ to impose palivizumab efficacy using data from Blanken *et al* 2013^[[24]](#endnote-25)^, Simoes *et al*. 2007^[[25]](#endnote-26)^ & Yoshihara *et al*. 2013^[[26]](#endnote-27)^ |
| **1–2** | 18.4% | 5.4% | 41.4% | 12.1% |  |
| **2–3** | 11.1% | 5.8% | 29.3% | 15.4% |  |
| **3–4** | 6.1% | 4.2% | 18.6% | 12.6% |  |
| **4–5** | 4.4% | 2.7% | 15.0% | 9.3% |  |
| **5–6** | 3.3% | 2.5% | 12.4% | 9.7% |  |
| **6–7** | 2.9% | 2.3% | 12.4% | 9.7% | Sigurs *et al*. 2000^[[27]](#endnote-28)^ adjusted to fit SPRING22 and modified as above |
| **7–13** | 2.3% | 1.5% | 17.4% | 11.0% | Sigurs *et al*. 2005^[[28]](#endnote-29)^ adjusted to fit SPRING22 and modified as above |
| **13–18** | 1.8% | 1.2% | 22.4% | 14.7% | Sigurs *et al*. 2010^[[29]](#endnote-30)^ adjusted to fit SPRING22 and modified as above |

RSV: respiratory syncytial virus; RSVH: RSV-related hospitalization. For infants with RSVH, long-term respiratory morbidity rates for up to 18 years are as displayed in Supporting Table 3. For infants with medically-attended RSV infection (MARI), the corresponding rates were taken from those labelled ‘No RSVH’ in Supporting Table 3. For infants without an RSV infection or an RSV infection not requiring any medical management were assumed to have some background respiratory morbidity for up to 6 years, using the same rates as for MARI.

**Figure S1: One-way deterministic sensitivity analysis (DSA) (±20%) for prophylaxed *versus* unprophylaxed infants covering 15 most sensitive variables for Korean guidelines (A) and IRST (B)**

**
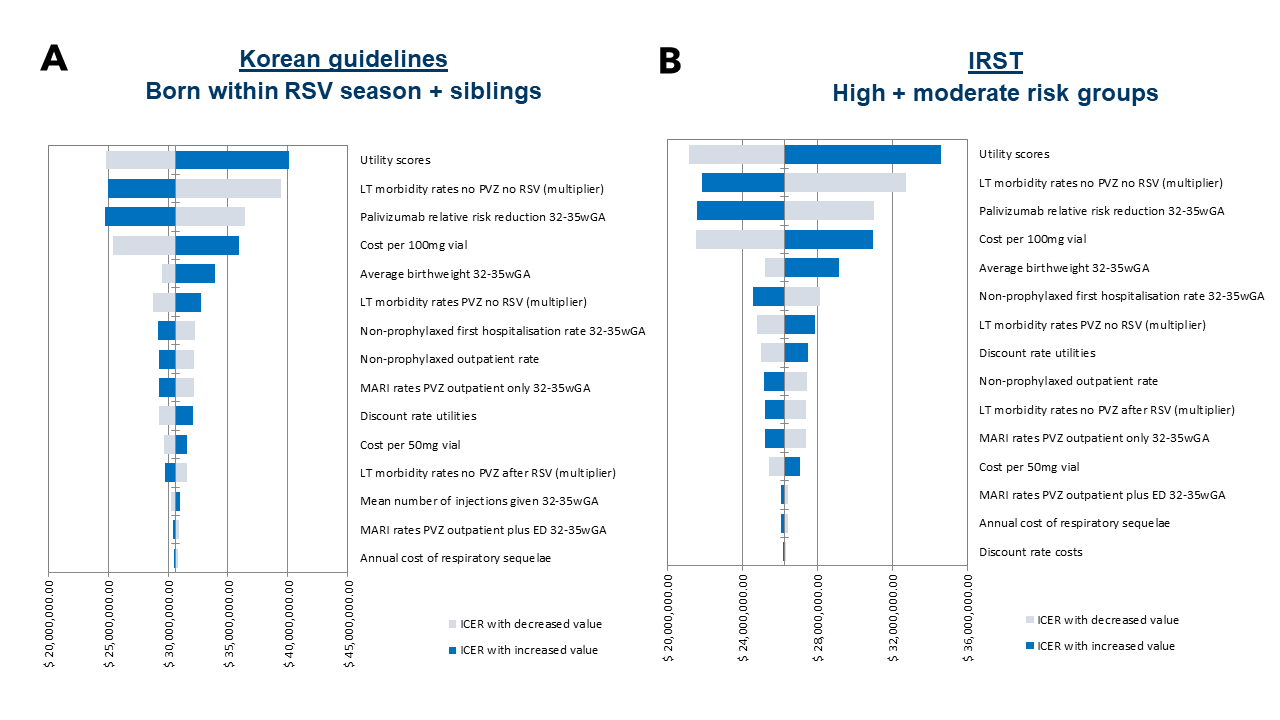
**

ED: emergency department; IRST: International Risk Scoring Tool; LT: long-term; MARI: medically-attended RSV infection; PVZ: palivizumab; RSV: respiratory syncytial virus; wGA: weeks’ gestational age

**References**

1. Blanken MO, Paes B, Anderson EJ, et al. Risk scoring tool to predict respiratory syncytial virus hospitalisation in premature infants. *Pediatr Pulmonol*. 2018; 53(5): 605-12. [↑](#endnote-ref-2)
2. Notario G, Vo P, Gooch K, et al. Respiratory syncytial virus-related hospitalization in premature infants without bronchopulmonary dysplasia: subgroup efficacy analysis of the IMpact-RS V trial by gestational age group. *Pediatric Health Med Ther*. 2014; 5: 43-8. [↑](#endnote-ref-3)
3. Weiner LB, Masaquel AS, Polak MJ, Mahadevia PJ. Cost-effectiveness analysis of palivizumab among pre-term infant populations covered by Medicaid in the United States. *J Med Econ*. 2012;15(5):997-1018. [↑](#endnote-ref-4)
4. Leidy NK, Margolis MK, Marcin JP, et al. The impact of severe respiratory syncytial virus on the child, caregiver, and family during hospitalization and recovery. *Pediatrics*. 2005; 115(6): 1536-46. [↑](#endnote-ref-5)
5. Greenough A, Alexander J, Burgess S, et al. Health care utilisation of prematurely born, preschool children related to hospitalisation for RSV infection. *Arch Dis Child*. 2004; 89(7): 673–8. [↑](#endnote-ref-6)
6. Chiou CF, Weaver MR, Bell MA, Lee TA, Krieger JW. Development of the multiattribute pediatric asthma health outcome measure (PAHOM). *Int J Qual Health Care*. 2005; 17(1): 23–30. [↑](#endnote-ref-7)
7. Wang D, Cummins C, Bayliss S, Sandercock J, Burls A. Immunoprophylaxis against respiratory syncytial virus (RSV) with palivizumab in children: a systematic review and economic evaluation. *Health Technol Assess*. 2008; 12(36): iii,ix-x,1-86. [↑](#endnote-ref-8)
8. Wang D, Bayliss S, Meads C. Palivizumab for immunoprophylaxis of respiratory syncytial virus (RSV) bronchiolitis in high-risk infants and young children: systematic review and additional economic modelling of subgroup analyses. *Health Technol Assess*. 2011; 15(5): iii-iv,1-124. [↑](#endnote-ref-9)
9. Ambrose CS, Anderson EJ, Simões EA, et al. Respiratory syncytial virus disease in preterm infants in the U.S. born at 32-35 weeks gestation not receiving immunoprophylaxis. *Pediatr Infect Dis J*. 2014; 33(6): 576-82. [↑](#endnote-ref-10)
10. Carbonell-Estrany X, Simões EAF, Dagan R, et al. Motavizumab for prophylaxis of respiratory syncytial virus in high-risk children: a noninferiority trial. *Pediatrics*. 2010; 125(1): e35–51. [↑](#endnote-ref-11)
11. Lim JS, Lim SW, Ahn JH, Song BS, Shim KS, Hwang IT. New Korean reference for birth weight by gestational age and sex: data from the Korean Statistical Information Service (2008-2012). *Ann Pediatr Endocrinol Metab*. 2014; 19(3): 146-53. [↑](#endnote-ref-12)
12. Kim YK, Song JY, Jang H, Kim TH, Koo H, Varghese L, Han E. Cost Effectiveness of Quadrivalent Influenza Vaccines Compared with Trivalent Influenza Vaccines in Young Children and Older Adults in Korea. *Pharmacoeconomics*. 2018; 36(12): 1475-90. [↑](#endnote-ref-13)
13. Park CS, Kang HY, Kwon I, Kang DR, Jung HY. [Cost-of-illness study of asthma in Korea: estimated from the Korea National Health insurance claims database]. *J Prev Med Public Health*. 2006; 39(5): 397-403. [↑](#endnote-ref-14)
14. Taxi Calculator. Taxi Rate Seoul [Cited 2024 August 01]. Available from: <https://www.taxi-calculator.com/taxi-rate-seoul/370>. [↑](#endnote-ref-15)
15. Statista. Average monthly salary of employees in South Korea from 2012 to 2023. Release date May 2024 [Cited 2024 August 01]. Available from: <https://www.statista.com/statistics/689751/south-korea-average-wage/>. [↑](#endnote-ref-16)
16. Mitchell I, Defoy I, Grubb E. Burden of Respiratory Syncytial Virus Hospitalizations in Canada. *Can Respir J.* 2017; 2017: 4521302. [↑](#endnote-ref-17)
17. Organisation for Economic Cooperation and Development (OECD). OECD Employment Outlook 2022 – Building Back More Inclusive Labour Markets. Korea. Release date September 2022 [Cited 2024 August 01]. Available from: <https://www.oecd-ilibrary.org/docserver/2776abcb-en.pdf?expires=1724838177&id=id&accname=guest&checksum=689C9F305FF8C95F635AC68A087E13B3>. [↑](#endnote-ref-18)
18. World Bank Group. Consumer price index (2010 = 100) – Korea, Rep [Cited 2024 August 01]. Available from: <https://data.worldbank.org/indicator/FP.CPI.TOTL?end=2022&locations=KR&start=2004>. [↑](#endnote-ref-19)
19. Statistics Canada. Consumer Price Index, monthly, not seasonally adjusted. Table: 18-10-0004-01. Release date: 2024-08-20 [Cited 2024 August 01]. Available from: <https://www150.statcan.gc.ca/t1/tbl1/en/tv.action?pid=1810000401>. [↑](#endnote-ref-20)
20. Organisation for Economic Cooperation and Development (OECD). Data Explorer. Purchasing Power Parities (PPP). South Korea [Cited 2024 August 01]. Available from: [https://data-explorer.oecd.org/vis?lc=en&df[ds]=DisseminateArchiveDMZ&df[id]=DF_DP_LIVE&df[ag]=OECD&av=true&pd=2020%2C2022&dq=CAN%2BKOR%2BOAVG%2BOECD.PPP...A&to[TIME_PERIOD]=false&vw=tb&lb=bt](https://data-explorer.oecd.org/vis?lc=en&df%5bds%5d=DisseminateArchiveDMZ&df%5bid%5d=DF_DP_LIVE&df%5bag%5d=OECD&av=true&pd=2020%2C2022&dq=CAN%2BKOR%2BOAVG%2BOECD.PPP...A&to%5bTIME_PERIOD%5d=false&vw=tb&lb=bt). [↑](#endnote-ref-21)
21. Narayan O, Bentley A, Mowbray K, et al. Updated cost-effectiveness analysis of palivizumab (Synagis) for the prophylaxis of respiratory syncytial virus in infant populations in the UK. *J Med Econ.* 2020; 23(12): 1640-52. [↑](#endnote-ref-22)
22. Carbonell-Estrany X, Pérez-Yarza EG, Sanchez García L, et al. Long-Term Burden and Respiratory Effects of Respiratory Syncytial Virus Hospitalization in Preterm Infants-The SPRING Study. *PLoS One.* 2015; 10(5): e0125422. [↑](#endnote-ref-23)
23. Sanchez-Luna M, Burgos-Pol R, Oyagüez I, et al. Cost-utility analysis of Palivizumab for Respiratory Syncytial Virus infection prophylaxis in preterm infants: update based on the clinical evidence in Spain. *BMC Infect Dis*. 2017; 17(1): 687. [↑](#endnote-ref-24)
24. Blanken MO, Rovers MM, Molenaar JM, et al. Respiratory syncytial virus and recurrent wheeze in healthy preterm infants. *N Engl J Med*. 2013; 368(19): 1791–9. [↑](#endnote-ref-25)
25. Simoes E, Groothuis JR, Carbonell-Estrany X, et al. Palivizumab prophylaxis, respiratory syncytial virus, and subsequent recurrent wheezing. *J Pediatr*. 2007; 151(1): 34–42. [↑](#endnote-ref-26)
26. Yoshihara S, Kusuda S, Mochizuki H, et al. Effect of palivizumab prophylaxis on subsequent recurrent wheezing in preterm infants. *Pediatrics* 2013; 132(5): 811–8. [↑](#endnote-ref-27)
27. Sigurs N, Bjarnason R, Sigurbergsson F, Kjellman B. Respiratory syncytial virus bronchiolitis in infancy is an important risk factor for asthma and allergy at age 7. *Am J Respir Crit Care Med*. 2000*;* 161(5): 1501-7. [↑](#endnote-ref-28)
28. Sigurs N, Gustafsson PM, Bjarnason R, et al. Severe respiratory syncytial virus bronchiolitis in infancy and asthma and allergy at age 13. *Am J Respir Crit Care Med.* 2005; 171(2): 137-41. [↑](#endnote-ref-29)
29. Sigurs N, Aljassim F, Kjellman B, et al. Asthma and allergy patterns over 18 years after severe RSV bronchiolitis in the first year of life. *Thorax*. 2010; 65(12): 1045–52. [↑](#endnote-ref-30)
